# Effects of gentaminoseleferon on blood parameters during treatment of *Mycoplasma dispar* respiratory infection in calves

**DOI:** 10.14202/vetworld.2020.2197-2202

**Published:** 2020-10-21

**Authors:** Mohammad Abed Alhussen, Hamdan Naef, Yury Anatolyevich Vatnikov

**Affiliations:** Department of Veterinary Medicine, Peoples’ Friendship University of Russia (RUDN University), 6 Miklukho-Maklaya Street, Moscow 117198, Russian Federation

**Keywords:** blood parameters, calves, gentaminoseleferon, *Mycoplasma dispar*, respiratory diseases

## Abstract

**Background and Aim:**

Respiratory diseases in young cattle are among the significant cattle pathologies that cause considerable economic damage globally. For the treatment of respiratory diseases, coformulated drugs that increase general nonspecific resistance, exhibit adaptogenic and anti-inflammatory properties, and normalize metabolic processes in animals are currently being used. The aim of our study was to investigate the therapeutic efficacy of the complex drug “gentaminoseleferon,” used in the treatment of respiratory diseases in calves, especially in *Mycoplasma dispar* infection.

**Materials and Methods:**

The animals were divided into three groups. Calves with the first clinical signs of respiratory pathology were randomly divided into two groups. The first experimental group (n=5) was intramuscularly injected with sulfetrisan at a dose of 5-10 mL/animal once per day for 7 days. The second experimental group (n=5) was given gentaminoseleferon at a dose of 1 mL/10 kg of body weight once per day for 7 days. The drugs were not used in the control group, the “healthy animals (n=5).” Blood samples were taken 10 days before and after treatment and compared between the experimental and healthy calves. The changes in the hematological and biochemical parameters of blood and serum were evaluated.

**Results:**

During the recovery process of animals in the experimental groups, a normalization of the hematological and biochemical parameters of blood and serum was noted. Interestingly, in calves of the second experimental group, an increase in the total serum protein content by 2.2% (p<0.05) was recorded in comparison with the first group. The second group, furthermore, showed an increase in Vitamins A, E, and C concentrations by 13.5% (p<0.05), 11.9% (p<0.005), and 15.1% (p<0.0005), respectively, as well as in zinc and iron concentrations by 4.1% (p<0.05) and 9.3% (p<0.0001), respectively. These findings indicate a more pronounced decrease in the inflammatory process in the respiratory system and intensive restoration of metabolism, thereby establishing the high therapeutic efficacy of gentaminoseleferon.

**Conclusion:**

Gentaminoseleferon was proven highly effective in the treatment of calves with respiratory illnesses and in restoring homeostasis in the organisms of animals after treatment, as indicated by the normalization of morphological and biochemical blood parameters with a reduction in the recovery time.

## Introduction

Respiratory diseases in calves are among the most significant cattle pathologies in the world [1]. The morbidity in calves due to respiratory diseses is about 30-65% annually and causes a mortality rate of 10-31% [2-4]. In addition, respiratory diseases hamper the growth rate of young animals, growth retardation, and dysfunction of the reproductive organs, which in sum causes significant economic damage [5].

*Mycoplasma dispar* is one of the most important agents causing respiratory disease in cattle [6,7] that are classified under genus *Mycoplasma* and class Mollicutes. *M. dispar* has a small genome size of 803 kbp [8], possesses no cell wall, and has a low guanine-cytosine ratio (28.5-29.3 mol%) [9]. *M. dispar* causes mild pneumonia in calves and mastitis in adult cattle. It is usually isolated from lungs and nasal swabs of pulmonary and healthy calves. *M. dispar* is very sensitive to culture *in vitro* and requires a special media. It is considered one of the slowest growing types of mycoplasmas [10]. Many studies have indicated that *M. dispar* is present in 50% of examined flocks and has coexisted with other bacterial agents, such as *Pasteurella multocida*, *Arcanobacterium pyogenes*, and *Mannheimia haemolytica* [11]. *M. dispar* in calves appears to be less immunogenic, and the vaccine, which is given by intramuscular injection (inoculations were antigen mixed with Freund’s complete or incomplete adjuvant), intra-tracheal (inoculations consisted of antigen in saline), or both, does not provide detectable protection against colonization, which can be at least partially pathogenic [12].

To increase immunity in young animals, it is recommended to use complex preparations as a form of treatment and prophylactic measures that contribute to the activation of metabolism, correction of immunity, and increase of nonspecific body resistance, among others [13-16].

One of such drug formulations is gentaminoseleferon, which contains gentamicin sulfate, aminoseleton, and a mixture of bovine recombinant α- and γ-interferon (INF) proteins. Gentamicin has a bactericidal effect against most Gram-positive and Gram-negative micro-organisms. The principle of action of gentamicin is its binding to the 30S subunit of bacterial ribosomes, impairing protein synthesis, and preventing the formation of tRNA and mRNA complexes, which leads to the erroneous reading of RNA and the formation of non-functional proteins in bacterial cells [17]. Aminoseleton is obtained by cryofractionation using biotechnology and represents a hydrophilic fraction of bovine spleen tissue, which contains a complex of biologically active substances such as amino acids, peptides, and nucleic acids. Aminoseleton increases the nonspecific resistance of the body, accelerates metabolism, and exhibits adaptogenic and antioxidant properties [18-20]. Bovine recombinant INFs implement rapid induction of the endogenous cytokine system and cellular and humoral immunity and exert anti-inflammatory and anti-stress effects. α-INF and γ-INF are synergistic, enhance the antibacterial effect of antibiotics, and reduce their possible suppressive effects [21,22].

The aim of the present study was to elucidate the therapeutic efficacy of the complex preparation gentaminoseleferon (developed at the Federal State Budgetary Scientific Institution [FSBSI], All-Russian Scientific Research Veterinary Institute of Pathology, Pharmacology and Therapy) in the treatment of *M. dispar* respiratory infections in calves.

## Materials and Methods

### Ethical approval

The study was conducted in accordance with the State program of Peoples’ Friendship University of Russia (RUDN University) after receiving approval from the Shared Research and Educational Center (SREC PFUR).

### Study location and period

The study was conducted at the research laboratory of the Department of Human and Animal Physiology at the Voronezh State University and the Department of Experimental Therapy in the All-Russian Scientific Research Veterinary Institute of Pathology, Pharmacology and Therapy, in the year 2019 (from 20 May to 11 August). The clinical studies were carried out on 3-5-month old red-mottled breed calves in a farm operated by Voronezhpishcheproduct Ltd., Novaya Usman, Voronezh Region.

### Animals and experimental design

Fifteen calves weighing from 75 to 88 kg were selected and divided into three groups. Calves with the first clinical signs of respiratory illness were randomly divided into two experimental groups (n=5 each). The calves in the first experimental group (n=5) were subjected to the treatment program adopted on the farm using sulfetrisan administered intramuscularly at 5-10 mL per animal once per day for 7 days. Meanwhile, the calves in the second experimental group (n=5) were intramuscularly injected with a new preparation, gentaminoseleferon, at 1 mL/10 kg of body weight, once per day for 7 days. This dosage was determined based on the previous pharmacological and clinical research conducted at the institute. Healthy calves (n=5) comprised the third group as the control and were not given any treatments.

### Clinical, bacteriological and molecular genetic examinations

The diagnosis was performed comprehensively based on the results of clinical and laboratory studies. We took into account the general indicators of health, such as body temperature, food intake, behavior, and habitus. The respiratory system was assessed by visual inspection (respiratory rate, nasal discharge, type of breathing, shortness of breath, dry, or wet spontaneous cough) and auscultation (increased or decreased volume of breathing sounds, rales, and signs of breathing difficulty). Materials collected for this study were blood, serum, and nasal cavity swabs. Bacteriological studies were carried out by culturing the pathogens on appropriate media using generally accepted methods at the FSBSI Research Center, All-Russian Scientific Research Veterinary Institute of Pathology, Pharmacology and Therapy. Molecular genetic studies of the biological materials were conducted through the real-time polymerase chain reaction (qPCR) using own commercial kits for M. dispar, infectious bovine rhinotracheitis virus, bovine viral diarrhea virus, and parainfluenza virus-3 detection in accordance with their instructions for use.

### Blood tests

To conduct hematological and biochemical studies, the blood samples were taken from both healthy and infected calves (experimental groups) 10 days before and after treatment. Hematological analysis was carried out on a ABX Micros 60 hematology analyzer using standard methods. Biochemical tests on blood serum were performed on a Hitachi-902 analyzer. During the experiment, the calves were monitored, the general health condition of the animals and weight gain were evaluated, the number of recovered animals and the duration of treatment and the timing of recovery were taken into account.

###  Statistical analysis

A comparative assessment of the average values of the measurements was carried out using analysis of variance using the statistical program StatPlus version 5 (AnalystSoft – USA).

## Results and Discussion

Respiratory diseases in young cattle cause inflammation that negatively impact the average daily weight gain and growth-to-feed ratio [23,24]. The cytokines released as a result of inflammation induce clinical symptoms, including fever, a sharp decrease in appetite, and, consequently, weight loss. Changes in some metabolic processes are also brought about, such as increased white blood cell count, decreased zinc, iron, vitamin, and other microelement serum concentrations [24-26]. A widespread prevalence of cattle mycoplasma in farms in the Russian Federation has been reported [27,28] where it is considered one of the most important agents in respiratory system illnesses in calves. Table-1 shows the spread of *M. dispar* in the Russian Federation in the years 2015-2018 [27].

**Table 1:** The prevalence of *Mycoplasma dispar* in the Russian Federation between 2015 and 2018.

Year	2015	2016	2017	2018
*Mycoplasma dispar* (%)	22.1	27.2	53.2	46.1

Bacteriological examination of nasal swabs collected from infected calves isolated *Staphylococcus aureus*, *Staphylococcus epidermidis*, *M. dispar*, and *P. multocida*, which are sensitive to gentamicin, tylosin, neomycin, norfloxacin, enrofloxacin, amoxicillin, ampicillin, doxycycline, and tetracycline. Molecular genetic analysis isolated the *M. dispar* genome, whereas genomes of the infectious bovine rhinotracheitis virus, bovine viral diarrhea virus, and parainfluenza virus-3 were not found.

The results of some hematological and biochemical parameters in calves during treatment with sulfetrisan are presented in Tables-2 and 3.

**Table 2:** Changes in some hematological parameters in calves during treatment with sulfetrisan (mean±SD).

Parameter	Healthy calves	Infected calves	

		Before treatment	After treatment
RBC (10^12^/L)	7.22±0.144	8.31±0.199**	7.85±0.268*
WBC (10^9^/L)	7.50±0.288	10.26±0.457**	8.03±0.410♦♦
Hb (g/L)	113.25±1.943	97.50±1.936***	108.25±0.559*♦♦

Hb=Hemoglobin, RBC=Red blood cell, WBC=White blood cell.

* p<0.02-0.05;

** p<0.001-0.005;

*** p<0.0002-0.0005 in relation to the parameters of healthy calves;

^♦^p<0.05;

♦♦ p<0.001-0.005 in relation to parameters before treatment

**Table 3:** Changes in some biochemical parameters in blood serum of calves during treatment with sulfetrisan (mean±SD).

Parameter	Healthy calves	Infected calves	

		Before treatment	After treatment
Vitamin A (μmol/L)	1.06±0.093	0.73±0.038**	0.89±0.025*♦♦
Vitamin E (μmol/L)	9.72±0.387	7.12±0.254***	8.62±0.097**♦♦♦
Vitamin C (μmol/L)	28.30±1.122	21.32±0.968***	25.34±0.650*♦♦
Total protein (g/L)	70.28±0.510	64.5±0.962**	69.58±0.459♦♦
Magnesium (mmol/L)	0.84±0.004	0.79±0.01***	0.81±0.007***
Zinc (μg/dL)	114.20±2.563	102.39±1.163***	107.88±1.773*♦♦
Iron (μg/dL)	195.66±0.937	157.20±3.549***	178.97±1.026***♦♦♦
Copper (μg/dL)	138.94±1.522	135.22±1.322*	136.64±1.123

* p<0.01-0.05;

** p<0.001-0.005;

*** p<0.0001-0.0005 in relation to parameters of healthy calves;

^♦^p<0.01-0.05;

♦♦ p<0.002-0.005;

♦♦♦ p<0.0001-0.0005 in relation to parameters before treatment

In sick calves of the first experimental group, the white blood cell count increased by 36.8% (p<0.001, indicating an inflammation), hemoglobin concentration decreased by 13.9% (p<0.0005), and the red blood cell count increased by 15.1% (p<0.005), apparently as a compensatory reaction due to hypoxemia, which may, in turn, lead to polycythemia [29]). Treatment with sulfetrisan increased hemoglobin concentration by 11.0% (p<0.001) and decreased the white blood cell count by 21.7% (p<0.005). When compared with healthy animals, these hematological parameters indicate an incomplete body recovery after infection.

In sick calves of the first experimental group, serum concentrations of Vitamins A, E, and C decreased by 31.1% (p<0.005), 26.7% (p<0.0005), and 24.7% (p<0.0005), respectively, whereas the serum concentrations of magnesium, zinc, iron, and copper decreased by 6% (p<0.0005), 10.3% (p<0.0005), 19.7% (p<0.0001), and 2.7% (p<0.05), respectively. Furthermore, the total protein level in serum decreased by 8.2% (p<0.001), which indicates a decrease in energy processes and insufficiency of protein-mineral metabolism against the background of the inflammatory process. This seems to occur as a result of the anorexia and, thus, the lack of intake of the necessary minerals and vitamins, the blood serum concentrations of which depend mainly on dietary intake [30]. Moreover, the decrease in the serum concentrations of vitamins and microelements is due to increase demand to repair the damage caused by acute or chronic inflammation [31]. After treatment with sulfetrisan, calves showed an increase in concentrations of Vitamin A by 21.9% (p<0.002), Vitamin C by 18.9% (p<0.002), and Vitamin E by 21.1% (p<0.0005). Blood serum concentrations of zinc, iron, and total protein also increased by 5.4% (p<0.005), 13.8% (p<0.0005), and 7.9% (p<0.002), respectively.

However, compared with those of healthy animals, Vitamins A, E, and C concentrations were lower by 16% (p<0.05), 11.3% (p<0.005), and 10.5% (p<0.05), respectively, whereas blood serum magnesium, zinc, and iron concentrations in blood serum were lower by 3.6% (p<0.0005), 5.5% (p<0.05), and 8.5% (p<0.0001), respectively, indicating incomplete recovery after disease.

The results of some hematological and biochemical parameters in calves during treatment with gentaminoseleferon are presented in Tables-4 and 5.

**Table 4:** Changes in some hematological parameters in calves during treatment with gentaminoseleferon (mean±SD).

Parameter	Healthy calves	Infected calves	

		Before treatment	After treatment
RBC (10^12^/L)	7.22±0.144	8.50±0.229**	7.60±0.316♦
WBC (10^9^/L)	7.50±0.288	10.80±0.505***	7.71±0.311♦♦
Hb (g/L)	113.25±1.943	95.88±3.461**	109.88±0.944♦♦

Hb=Hemoglobin, RBC=Red blood cell, WBC=White blood cell.

*p<0.02-0.05;

** p<0.002;

*** p<0.0001-0.0005 in relation to the parameters of healthy calves;

♦ p<0.05;

♦♦ p<0.001-0.005 in relation to parameters before treatment

**Table 5:** Changes in some biochemical parameters in blood serum of calves during treatment with gentaminoseleferon (mean±SD).

Parameter	Healthy calves	Infected calves	

		Before treatment	After treatment
Vitamin A (μmol/L)	1.06±0.093	0.72±0.04**	1.01±0.059♦♦♦
Vitamin E (μmol/L)	9.72±0.387	7.34±0.266***	9.65±0.359♦♦♦
Vitamin C (μmol/L)	28.30±1.122	21.44±0.535***	29.16±0.5♦♦♦
Total protein (g/L)	70.28±0.510	63.52±0.734***	71.10±0.503♦♦♦
Magnesium (mmol/L)	0.84±0.004	0.79±0.009***	0.82±0.07
Zinc (μg/dL)	114.20±2.563	102.30±1.479***	112.26±1.674♦♦♦
Iron (μg/dL)	195.66±0.937	156.15±5.617***	195.61±0.928♦♦♦
Copper (μg/dL)	138.94±1.522	134.96±0.690*	137.40±1.352

* p<0.01-0.05;

** p<0.001-0.005;

*** p<0.0001-0.0005 in relation to parameters of healthy calves;

^♦^p<0.01-0.05;

^♦♦^p<0.002-0.005;

♦♦♦ p<0.0001-0.0005 in relation to parameters before treatment

The inflammatory process in calves of the second experimental group was indicated by a decrease in hemoglobin concentration by 15.3% (p<0.002), an increase in the red blood cell count by 17.7% (p<0.002), and an increase in the white blood cell count by 44.0% (p<0.0005). After treatment with gentaminoseleferon, hemoglobin levels increased by 14.6% (p<0.005), the red blood cell count decreased by 10.5% (p<0.05), and white blood cell count decreased by 28.6% (p<0.001).

In the calves from the second group, Vitamins A, E, and C concentrations in blood serum decreased by 32.1% (p<0.005), 24.5% (p<0.0005), and 24.2% (p<0.0001), respectively, whereas magnesium, zinc, iron, and copper concentrations decreased by 6% (p<0.0001), 10.4% (p<0.0005), 20.2% (p<0.0001), and 2.9% (p<0.05), respectively. Furthermore, the total protein level decreased by 9.6% (p<0.0001), which indicated insufficient metabolic activity during the respiratory infection.

During recovery after treatment with gentaminoseleferon, Vitamins A, C, and E concentrations increased by 40.3% (p<0.0005), 36% (p<0.0001), and 31.5% (p<0.0001), respectively, whereas zinc, iron, and total protein concentrations increased by 9.7% (p<0.0005), 25.3% (p<0.0001), and 11.9% (p<0.0005), respectively. This indicated a decline in the inflammatory process and normalization of metabolism.

It should be noted that the use of gentaminoseleferon, as compared with sulfetrisan, contributed to an increase in the total protein content by 2.2% (p<0.05), and in Vitamins A, E, and C concentrations by 13.5% (p<0.05), 11.9% (p<0.005), and 15.1% (p<0.0005), respectively. Furthermore, serum concentrations of zinc and iron increased by 4.1% (p<0.05) and 9.3% (p<0.0001), respectively. The improvements in these parameters indicated a more pronounced decrease in the inflammatory process and intensive restoration of protein-energy metabolism in calves in the second experimental group. The ability of components of gentaminoseleferon to influence the indicators of nonspecific resistance is confirmed by previous studies [18,22].

The results of the therapeutic efficacy of sulfetrisan and gentaminoseleferon in the treatment of respiratory diseases in calves are presented in Table-6.

**Table 6:** Therapeutic efficacy of sulfetrisan and gentaminoseleferon in treatment of respiratory diseases of calves.

Indicator	Sulfetrisan	Gentaminoseleferon
The number of animals in the group (animal)	5	5
Duration of treatment (days)	7	7
Number of recovered animals (%)	4 (80)	5 (100)
Number of dead animals (%)	0 (0)	0 (0)
Remained sick animals (%)	1 (20)	0 (0)
Calves weight at the beginning of the experiment (kg)	83.50±0.866	84.88±1.025
Calves weight at the end of the experiment (kg)	88.86±0.996	91.55±0.944*
Average of daily gain (g)	412±10.2	513±22.7*
Recovery duration (days)	14.13±0.854	10.88±0.398*
Therapeutic efficacy %	80.0	100.0

* p<0.01-0.002 in relation to indicators of calves treated with sulfetrisan.

Based on the data gathered, gentaminoseleferon showed high therapeutic efficacy (100%) in the treatment of respiratory diseases in calves and 0.20% higher than the comparison drug. At the same time, the recovery time was 23.3% (p<0.01) shorter and the average daily weight gain upon recovery was 24.5% (p<0.002) higher.

## Conclusion

Our results show that, due to its components, gentaminoseleferon exhibits high therapeutic efficacy in the treatment of calves with respiratory diseases and helps reduce their recovery time. The drug provides an intensive restoration of protein-energy metabolism and a pronounced decrease in the inflammatory process, as proved by the normalization of morphological and biochemical blood parameters. Moreover, the drug has the ability to improve the general metabolism processes in calves, represented by improved levels of vitamins and microelements in the blood serum. Further studies of the mechanism of action of the drug on a large scale, as well as its introduction into calf breeding technology, are a promising direction for pharmacocorrection of respiratory pathologies.

## Authors’ Contributions

MAA and HN carried out the experiments, analyzed and interpreted of data, and drafted the manuscript. YAV supervised the project, and revised the manuscript. All authors have checked and approved the final version of the manuscript.

## References

[1] Murray G.M, More S.J, Clegg T.A, Earley B, O'Neill R.G, Johnston D, Gilmore J, Nosov M, McElroy M.C, Inzana T.J, Cassidy J.P (2018). Risk factors associated with exposure to bovine respiratory disease pathogens during the peri-weaning period in dairy bull calves. BMC Vet. Res.

[2] Constable P, Hinchcliff K.W, Done S, Gruenberg W (2016). Veterinary Medicine:A Textbook of the Diseases of Cattle, Horses, Sheep, Pigs and Goats Two-Volume Set.

[3] Taylor J.D, Fulton R.W, Lehenbauer T.W, Step D.L, Confer A.W (2010). The epidemiology of bovine respiratory disease:What is the evidence for predisposing factors?. Can. Vet. J.

[4] Dubrovsky S.A, van Eenennaam A.L, Karle B.M, Rossitto P.V, Lehenbauer T.W, Aly S.S (2019). Epidemiology of bovine respiratory disease (BRD) in preweaned calves on California dairies:The BRD 10K study. J. Dairy Sci.

[5] Louie A.P, Rowe J.D, Love W.J, Lehenbauer T.W, Aly S.S (2018). Effect of the environment on the risk of respiratory disease in preweaning dairy calves during summer months. J. Dairy Sci.

[6] Mosier D (2014). Review of BRD pathogenesis:The old and the new. Anim. Health Res. Rev.

[7] Tortorelli G, Gaeta N.C, Ribeiro B.L.M, Marques L.M, Timenetsky J, Gregory L (2017). Evaluation of *Mollicutes* microorganisms in respiratory disease of cattle and their relationship to clinical signs. J. Vet. Intern. Med.

[8] Herrmann R (1992). Genome structure and organization. Mycoplasmas: Molecular Biology and Pathogenesis. American Society for Microbiology.

[9] Nicholas R, Ayling R, McAuliffe L (2008). *Mycoplasma* Diseases of Ruminants.

[10] Chen S, Hao H, Yan X, Liu Y, Chu Y (2019). Genome-wide analysis of *Mycoplasma* dispar provides insights into putative virulence factors and phylogenetic relationships. G3 (*Bethesda*).

[11] Bednarek D, Szymańska-Czerwińska M, Dudek K, Perez-Marin C.C (2012). Bovine respiratory syndrome (BRD) etiopathogenesis, diagnosis and control. A Bird's-Eye View of Veterinary Medicine.

[12] Barrett D.C (2000). The calf pneumonia complex-treatment decisions. Cattle Pract.

[13] Abramov V.E, Balyshev A.V, Kashkovskaya L.M, Safarova M.I (2017). Lexoflon a new remedy for the treatment of calves with respiratory diseases. Vet. Med.

[14] Polozyuk O.N (2018). Treatment of calf bronchopneumonia in conditions of livestock complexes. Bull. Don State Agrarian Univ.

[15] Chernitskiy A.E, Shabunin S.V (2017). Prevention of respiratory diseases in the newborn calves with decreased viability. Vet. Med.

[16] Pardon B, Callens J, Maris J, Allais L, van Praet W, Deprez P, Ribbens S (2020). Pathogen-specific risk factors in acute outbreaks of respiratory disease in calves. J. Dairy Sci.

[17] Aleksandrovskiy Y.A, Vyshkovskiy G.L, Return Merchandise Authorization (2012). The Register of Medicines of Russia. Encyclopedia of Medicines: Annual Collection Vedanta Moscow.

[18] Shakhov A.G, Shabunin S.V, Vostroilova G.A, Bliznetsova G.N, Sashnina L.Y, Kantorovich Y.A (2017). Antioxidant status in albino rats vaccinated against salmonellosis under chronic action of T-2 toxin and its correction by aminoseleton. Russ. Agric. Sci. J.

[19] Savrasov D.A, Parshin P.A, Vatnikov Y.A, Kulikov E.V, Popova I.A, Nikishov A.A, Petrov A.K, Petukhov N.V, Molchanova M.A (2019). Correction of the immune status of cows by using aminoseleton during the dry period for prevention of antenatal calf hypotrophy. J. Anim. Health Prod.

[20] Miroshnichenko A.G, Brukhanov V.M, Gossen I.Y, Perfilyev V.Y (2016). Antioxidants capabilities in correction of gentamicin-induced nephrotoxicity in experimental infection. Kazan Med. J.

[21] Altynbekov O.M, Andreeva A.V (2019). The effect of the drug bovine recombinant interferon and immunal on the accumulation of specific antibodies to causative agents of associative infections in the blood of calves. Bull. Bashkir State Agrarian Univ.

[22] Zaitseva A.V, Prokulevich V.A, Dremach G.E, Zaitseva V.V (2019). The effect of bovine interferon in the composition of enrofloxavetferon-B on the content of specific proteins in the blood serum of calves. Viciebsk State Acad. Vet. Med.

[23] Gardner B.A, Dolezal H.G, Bryant L.K, Owens F.N, Smith R.A (1999). Health of finishing steers:Effects on performance, carcass traits, and meat tenderness. J. Anim. Sci.

[24] Gifford C.A, Holland B.P, Mills R.L, Maxwell C.L, Farney J.K, Terrill S.J, Step D.L, Richards C.J, Burciaga Robles L.O, Krehbiel C.R (2012). Growth and development symposium:Impacts of inflammation on cattle growth and carcass merit. J. Anim. Sci.

[25] Cray C, Zaias J, Altman N.H (2009). Acute phase response in animals:A review. Comp. Med.

[26] Gruys E, Toussaint M.J.M, Niewold T.A, Koopmans S.J (2005). Acute phase reaction and acute-phase proteins. J. Zhejiang Univ. Sci.

[27] Abed Alhussen M, Nesterov A.A, Kirpichenko V.V, Yatsentyuk S.P, Sprygin A.V, Byadovskaya O.P, Kononov A.V (2020). Bovine mycoplasmosis occurrence on livestock farms in the Russian Federation for 2015-2018. Vet. Sci. Today.

[28] Sukhinin A.A, Makavchik S.A, Kuzmin V.A, Fogel L.S, Orekhov D.A, Karpenko L.Y, Kan F.L (2017). Methodical Guidelines for Livestock and Poultry Mycoplasmoses Prevention and Eradication. State Academy of Veterinary Medicine, Saint Petersburg.

[29] Faez F, Abdinasir Y.O, Lawan A, Zunita Z, Rasedee A, Mohd Z.S, Abdul A.S (2013). Haematological and biochemical alterations in calves following infection with *Pasteurella multocida* Type B:2, bacterial lipopolysaccharide and outer membrane protein immunogens (OMP). Asian J. Anim. Vet. Adv.

[30] Rowlands G.J (1980). A review of variations in the concentrations of metabolites in the blood of beef and dairy cattle associated with physiology, nutrition and disease, with particular reference to the interpretation of metabolic profiles. World Rev. Nutr. Diet.

[31] Caram L.M.O, Amaral R.A.F, Ferrari R, Tanni S.E, Correa C.R, Paiva S.A.R, Godoy I (2015). Serum Vitamin A and inflammatory markers in individuals with and without chronic obstructive pulmonary disease. Mediators Inflamm 2015.

